# Psychiatric morbidity and socio-occupational dysfunction in residents of a drug rehabilitation centre: challenges of substance misuse management in a Bruneian context^†^

**DOI:** 10.1192/pb.bp.113.046300

**Published:** 2015-10

**Authors:** Hilda Ho, Anddy Maz Adanan, Radiah Omar

**Affiliations:** 1 Ministry of Health, Brunei

## Abstract

**Aims and method** In 2011, a psychiatric clinic was started in Pusat Al-Islah, a drug rehabilitation centre. Our aim was to record self-reported socio-occupational dysfunction and patterns of drug misuse and to evaluate the usefulness of a psychiatric screening tool. A two-phased approach using the Self-Reporting Questionnaire (SRQ) and the Mini International Neuropsychiatric Interview (MINI) was used to examine the rates of psychiatric diagnoses.

**Results** Methamphetamine was the most commonly misused substance in 94.5% of residents. High levels of socio-occupational dysfunction were reported. In total, 5.5% met criteria for major depressive disorder, 4.8% for lifetime psychotic disorder and 11.5% for suicidal ideation. In addition, 13.3% reported previous untreated mental health problems.

**Clinical implications** A screening tool such as the SRQ can be used to identify those needing further psychiatric assessment. Interventions to address amphetamine misuse and associated socio-occupational dysfunction are required. Societal views and legislation influence the management of substance misuse problems in Brunei.

Pusat Al-Islah is a residential drug rehabilitation centre run by the Narcotics Control Bureau (NCB) in Brunei, a small country in Southeast Asia. Residents are sent here after being convicted of drug use offences and testing positively for illegal substances. This is a prison diversion scheme that aims to rehabilitate drug misusers and reduce the rates of reoffending by offering a residential rehabilitation programme and post-release supervision. A small number are voluntary admissions. Treatment is based on a therapeutic community model and residents stay for up to 30 months. There is occupational training, Islamic religious lessons and counselling. A general practitioner and primary care nurse provide primary healthcare. In 2011, a psychiatric clinic was started. This was an opportunity to examine psychiatric morbidity among residents. The high prevalence of psychiatric morbidity in correctional and drug-misusing populations has been highlighted elsewhere, but there is no existing data for Brunei. This is a group who can be difficult to engage in treatment. Admission into the centre is an opportunity to screen for and treat any significant illness. Psychiatric morbidity is likely to have an impact on rehabilitation. Those individuals with mental disorders are more vulnerable to the adverse effects of imprisonment and are at higher risk of suicide.^1^ They may have difficulty adhering to rehabilitation programmes.^2^ We hypothesised that there would be a high level of psychiatric morbidity and socio-occupational dysfunction in this population. We also evaluated the usefulness of a psychiatric screening tool. Finally, we consider the challenges facing substance misuse management in the Bruneian context.

## Method

This was a cross-sectional study. Ethical approval was obtained from the Research & Ethics Committee, Ministry of Health, Brunei Darussalam. Those who were residing in Pusat Al-Islah on 1 January 2012 or admitted by 31 March 2012 were approached to take part in phase 1 of the project after giving written informed consent. The interviews were conducted by three researchers, a consultant forensic psychiatrist (H.H.), a doctor training in psychiatry (A.M.A.), and a senior psychiatric nurse (R.O.).

### Phase 1

All residents were interviewed using the Self-Reporting Questionnaire-24 (SRQ-24)^3^ between January and June 2012. This is a tool that has been shown to have good sensitivity and specificity for identifying psychiatric morbidity in primary healthcare settings.^3^ As Malay is the main language spoken in Brunei, we used a Malay language version that has been used to examine psychiatric morbidity in a Malay-speaking population.^4^ Questions 1–20 relate to neurotic symptoms and questions 21–24 to psychotic symptoms. Those who scored above a cut-off score of 6 and above for the first 20 questions and/or 1 and above for the last 4 questions, were entered into phase 2 of this study. This cut-off has been suggested to have the best ‘trade-off’ between sensitivity and specificity. Demographic details, educational and employment history, drug misuse history, psychiatric history, self-reported violence and offending history were also obtained. Data regarding socio-occupational function were obtained using an open questioning style. Any participant thought to require clinical assessment was referred to the psychiatric clinic.

### Phase 2

Residents proceeding to phase 2 were interviewed using the Malay version of the Mini International Neuropsychiatric Interview (MINI, version 6.0).^5^ The MINI is a short semi-structured diagnostic interview for DSM-IV^6^ and ICD-10^7^ psychiatric disorders. It has been shown to have good validity and interrater reliability.^5^ The Malay version has been shown to have good reliability in diagnosing major depressive disorder and generalised anxiety disorder in a Malaysian community setting.^8^ The sections coding for the following groups of disorders were used: major depressive episode, suicidality, manic and hypomanic episodes, psychotic disorders and mood disorder with psychotic features, generalised anxiety disorder. The prevalence of these disorders was the main focus of the study as they are common treatable causes of psychiatric morbidity. All interview records were checked by the consultant forensic psychiatrist (H.H.) for accuracy.

### Statistical analyses

Data were analysed using the Statistical Package for the Social Sciences, SPSS, version 16 for Windows.

## Results

### Demographics

In total, 195 residents were approached to take part and 165 residents (140 (84.8%) men and 25 (15.2%) women) consented and were interviewed for phase 1. Their mean age was 33.1 years (range 18–55, s.d. = 7.6). The mean length of stay before the interview was 14.8 months (range 1–33, s.d. = 9.7). A total of 155 (93.9%) were admitted under an order of the Courts or the Minister of Home Affairs; 10 (6.1%) were voluntary admissions. Of participants, 161 (97.6%) had been born in Brunei; 91 (55.2%) were married, 95 (57.6%) had children under the age of 18 years and 57 (34.5%) were unemployed at the time of admission. Well over half, 101 (61.2%) had not completed secondary school. All residents who declined consent were men.

### Index offence

In total, 148 (89.7%) were admitted for a drug use offence alone, 16 (9.7%) were admitted for drug dealing in addition to a drug use offence. Data were missing for 1 (0.6%) resident.

### Drug misuse history

The mean age of starting drug misuse was 19.8 years (range 9–46, s.d. = 7.1). The mean time period from starting drug use to the interview was 13.7 years (range 1–35, s.d. = 7.1). Methamphetamines were the most commonly misused substance. The drugs misused are described in Table 1. The majority of residents reported using drugs at least once a week and experienced some physical or psychological discomfort after a period of abstinence (Table 2).

**Table 1:** Type of substance misuse (*n* = 165)

Substance misused	*n* (%)
Crystal methamphetamine	159 (96.4)

Main drug misused	152 (92.1)

Other amphetamine/stimulants	23 (13.9)

Cannabis	61 (37.0)

Cough medicine	52 (31.5)

Solvents	16 (9.7)

Opiates	8 (4.8)

‘Pills’	64 (38.8)

Steroids	2 (1.2)

Intravenous drug misuse^a^	7 (4.2)

Alcohol problem^b^	102 (61.8)

a. All methamphetamines.

b. Residents who reported that they drank heavily or had an alcohol misuse problem.

**Table 2:** Frequency of drug misuse, and symptoms after abstinence (*n* = 165)

	*n* (%)
Frequency of drug use	
Daily	72 (43.6)
At least twice a week	49 (29.7)
Once a week	16 (9.7)
1–3 times a month	24(14.5)
Less than once a month	3 (1.8)

Symptoms after abstinence	
Experienced physical (shaking, sweating, sleep disturbance) or psychological (irritability, craving, anxiety) discomfort	115 (69.7)

### Psychiatric history

Of participants, 29 (17.6%) reported previous contact with psychiatric services, 9 (5.5%) residents were taking psychiatric medication (antidepressant, antipsychotic or mood stabiliser). In total, 13 (7.9%) had previous admissions for psychiatric treatment, 5 (3.0%) had received involuntary treatment under the Lunacy Act.^9^ Twenty-two participants (13.3%) reported experiencing mental health problems in the past, for which they had not sought treatment. There were 16 (9.7%) who reported experiencing previous suicidal ideation. In addition, 19 (11.5%) reported having a first- or second-degree relative with mental health problems. Psychiatric case-notes were found for 26 (15.8%) residents. When these were examined for recorded diagnoses, 13 (7.9%) had a psychotic illness (schizophrenia or acute psychotic episode), 9 (5.5%) had a depression, 1 (0.6%) had bipolar disorder, 2 (1.2%) had personality disorder and 1 (0.6%) had a child and adolescent mental disorder, unspecified. A total of 13 (7.9%) were recorded to have been non-adherent with treatment. Also, 9 (5.5%) participants had previous self-harm documented.

### Forensic history

In total, 28 (17.0%) had previous admissions to Pusat Al-Islah; 61 (37.0%) had served at least one prison sentence; 20 (12.1%) had been convicted of an previous offence without a prison sentence, 39 (23.6%) had been remanded by the police but released without charge or conviction. Also, 41 (24.8%) reported performing previous physical violence such as assault.

### Socio-occupational function

Of all the participants, 75 (45.5%) reported problems with their educational or work performance attributed to drug misuse. A total of 68 (41.2%) reported financial problems, 98 (59.4%) reported family relationship problems and 22 (23.2% of those with children <18 years old) reported having difficulty caring for their children adequately. Sixty participants (36.4%) reported offending behaviour related to their drug misuse. Also, 77 (46.7%) had a first-degree relative (parent, child or sibling) who misused drugs and 14 (8.5%) had a spouse or partner who misused drugs.

### SRQ scores

The mean score for questions 1–20 was 3.6 (range 0–18, s.d. = 3.9) and for questions 21–24 was 0.25 (range 0–3, s.d. = 0.7). A total of 40 (24.2%) residents, 31 men and 9 women, met criteria for entry into phase 2. When means were compared using the independent *t*-test, no significant differences were found between the phase 1 and phase 2 groups for age (32.2 v. 33.4 years, P = 0.386), duration of drug misuse problem (13.75 v. 13.5 years, P = 0.458) and age of starting drug misuse (20.1 v. 18.9 years, P = 0.357).

### Phase 2

Interviews occurred after phase 1 was completed, between August and October 2012. Of the 40 residents who were eligible for entry into phase 2, 10 residents had been discharged and were invited for interview, however, 7 discharged residents did not respond to the invitation for a second interview, 1 resident was not cooperative. Therefore, 32 participants (23 men (71.9%) and 9 women (28.1%)) were interviewed.

In total nine (5.5%) met criteria for major depressive disorder (two ‘current’, six ‘previous’ and one ‘current and previous’) of whom three (1.8%) met criteria for major depressive disorder with psychotic features (‘current’, ‘previous’ or ‘current and previous’). Three (1.8%) participants met criteria for previous manic episode, three (1.8%) met criteria for hypomanic episode (‘current’ or ‘previous’) and six (3.6%) met criteria for previous hypomanic symptoms. There were three (1.8%) participants who met criteria for bipolar I disorder (‘current’ or ‘previous’), three (1.8%) met criteria for bipolar II disorder (‘previous or current and previous’) and five (3.0%) met criteria for ‘uncategorised bipolar disorder’. Eight (4.8%) met criteria for lifetime psychotic disorder, of whom five (3.0%) met criteria for current psychotic disorder. There was one individual (0.6%) who met criteria for current generalised anxiety disorder. A total of 19 (11.5%) met criteria for suicidal ideation (15 at ‘low level’, 1 ‘medium level’ and 3 ‘high level’) (Table 3). Eight (4.8%) had more than one diagnosis.

**Table 3:** 

Mini International Neuropsychiatric Interview (MINI) diagnosis	*n* (%)
Major depressive disorder	9 (5.5)

Major depressive disorder with psychotic features	3 (1.8)

Manic episode	3 (1.8)

Hypomanic episode	3 (1.8)

Hypomanic symptoms	6 (3.6)

Bipolar I disorder	3 (1.8)

Bipolar II disorder	3 (1.8)

Uncategorised bipolar disorder	5 (3.0)

Lifetime psychotic disorder	8 (4.8)

Current psychotic disorder	5 (3.0)

Current generalised anxiety disorder	1 (0.6)

Suicidal ideation	19 (11.5)

>1 diagnosis	8 (4.8)

## Discussion

### Screening and diagnostic tools

The SRQ is easy and quick to use. The interviewers found that questions 1–20, which asked about depressive and neurotic symptoms, were more easily understood than questions 21–24, which asked about psychotic symptoms. In particular, question 22 referring to grandiosity was often misunderstood. It was concluded that the first 20 questions would be a useful screening tool for depressive and neurotic disorders, whereas the psychosis questions should be amended. Of the 40 residents who entered phase 2, 24 (60%) were found to have a diagnosis and/or suicidal ideation when interviewed with the MINI. Thus, this method of identifying patients with psychiatric disturbance appeared to have produced a high ‘yield’. The MINI, although straightforward to use as a diagnostic tool was time-consuming to administer. We concluded that the SRQ could be used to screen residents on admission, and those scoring above the cut-off referred for further assessment.

### Rates of mental disorders

Although the high prevalence of psychiatric morbidity in correctional populations has often been highlighted in Western countries,^10-13^ there is less data available in non-Western countries.^14^ High prevalence of mental disorders have been found in an Iranian prison population.^15^ Similarly, high rates of psychiatric morbidity were found in individuals who misused methamphetamine detained in Taiwan.^16^ This is the first investigation of psychiatric morbidity in a drug-misusing population in Brunei. Major depressive disorder was the most common diagnosis and was observed in 5.5% of the sample, similar to the Taiwanese finding. Lifetime psychotic disorder was found in 4.8% of residents, 3.0% of whom had a current psychotic disorder. Although we attempted to exclude psychotic symptoms directly associated with substance misuse, it is possible that some findings may have been methamphetamine psychosis rather than a primary psychotic disorder. Bipolar I disorder was observed in 1.8% of residents and bipolar II disorder was similarly observed in 1.8% of residents. The rates of psychotic^17^ and bipolar disorders^18^ appeared to be higher than in the general population, although no epidemiological data exist for the prevalence of mental disorders in Brunei.

### Previous psychiatric disturbance and socio-occupational dysfunction

We found substantial rates of psychiatric morbidity. Our results also indicate substantial socio-occupational dysfunction, violence and offending behaviour. Given that these were self-reported, it is likely that they were underestimated. These findings emphasise the need for psychiatric, psychological, behavioural and socio-occupational interventions.

### Pattern of drug misuse

Crystal methamphetamine is commonly available in Southeast Asia. It is locally known as ‘syabu’ and was the most common drug of misuse found in this sample. The first case of crystal methamphetamine misuse was reported to the NCB in 1993. Since then its use has spread across the country, affecting many lives and families. Drug supplies are manufactured abroad and transported across the border from neighbouring countries, often by land or water routes. Southeast Asia has a higher consumption of amphetamine-type substances compared with the global average. The neighbouring countries of Thailand, Malaysia, Cambodia and Indonesia have seized record high amounts of crystalline methamphetamine in the past few years. Annually, hundreds of illicit synthetic drug manufacturing facilities have been seized in the region over the same period.^19^ The availability of crystal methamphetamine is likely to contribute significantly to its popularity in Brunei. In contrast, the country has not had a widespread heroin misuse problem, avoiding the hazards associated with intravenous drug misuse. This pattern of drug misuse should inform national drug treatment and rehabilitation strategies. Substitute prescription such as methadone programmes used to treat heroin addiction more common elsewhere, cannot be usefully applied here.

We found that the rate of self-reported alcohol problems in this population was very high (61.8%). This may reflect the conservative Islamic view regarding alcohol use in Brunei, which may lower the threshold for viewing alcohol consumption as problematic and increase the likelihood of problems associated with its use.

### Societal views of drug and alcohol use

Societal views regarding drug and alcohol use are generally very conservative. This is reflected in the heavy penalties imposed on those convicted of drug misuse, dealing and trafficking offences.^20^ More than two-thirds of the population is Muslim and it is the official religion of the state. It is expected that alcohol should not be consumed at all by Muslims. The sale of alcohol is banned, and only non-Muslim adults are allowed to take controlled quantities into the country for personal use. Public drinking is not allowed. The phased introduction of the Syariah Penal Code,^21^ which began in April 2014, further increases the prospects of heavy punishment for those who contravene the strict laws relating to alcohol use. Under this new law, drinking alcohol is an offence for Muslims. Public drinking both in the country and abroad, providing alcohol to a Muslim and abetment of a Muslim to consume alcohol are listed as punishable offences for non-Muslims. Those convicted of alcohol offences may be fined, imprisoned and punished with whipping.

### Treatment for alcohol and drug misuse

Many people are reluctant to admit their problems or seek help, although individuals can present themselves to psychiatric services. Pharmaceutical preparations such as disulfiram and naltrexone are available for the treatment of alcohol misuse. Structured community alcohol or drug misuse treatment programmes run either by medical services or the voluntary sector, are not available. There is limited open discussion about drug and alcohol misuse problems. The cause and impact of these problems locally and treatment and rehabilitation are areas that have received little research attention. Rehabilitation in Pusat Al-Islah, similar to other rehabilitation and training schemes in the country, places a strong emphasis on Islamic religious counselling. Psychological and behavioural treatment programmes require much development.

Our clinical experience working in psychiatric treatment facilities suggest that many individuals with substance misuse problems also have mental health problems requiring multidisciplinary care. The government provides largely hospital-based psychiatric services. Community, subspecialist and multidisciplinary services are limited although there have been some recent developments. New mental health legislation, in the form of the Mental Health Order 2014, has been approved. This legislation is designed to ensure the appropriate care of people with mental disorders and is scheduled to be implemented on 1 November 2014. The development of multisector partnerships is crucial to fully addressing the complex needs of this population.

### Limitations

The two-phase design of this study may have reduced the accuracy of the results. Residents with a current diagnosis in phase 1 may have recovered before the phase 2 interview. However, the MINI allows for previous and lifetime diagnoses to be coded. The Malay translations of both tools may have limited validity in this population as the Malay dialect spoken in Brunei is slightly different to standard written Malay. The MINI interviewers were not masked to the diagnoses or treatment received by participants. This study depended on self-reporting of socio-occupational dysfunction as we were unable to obtain official reports. It is likely that the true prevalence of psychiatric disorders and socio-occupational dysfunction were higher given the limitations. The sample size in this study was small, with only 32 patients being finally assessed in phase 2. This may have affected the accuracy of our findings. We suggest that any future study should screen a larger sample of new residents on admission, with the diagnostic interview conducted as soon as individuals with mental disorders are identified. This would measure the incidence rates of mental disorders more accurately.

In conclusion, this is the first study to examine psychiatric morbidity, pattern of drug misuse and socio-occupational dysfunction in a Bruneian population of drug misusers. It highlights the treatment and intervention needs of this high-risk group and the challenges faced locally.
